# 

**Published:** 1895-10-26

**Authors:** 


At a meeting of the Board of Delegates of the Hospital Saturday Fund held last week, it was announced that the collection for 1895 amounted to £11,427 17s. 6d., viz.: Workshop collection, £6,948 6s. 5d.; street collection, £4,379 9s. 4d. (being a slight decrease upon last year), and miscellaneous, £100 1s. 9d.; while the expenditure was £1,778 9s. 9d. The sum contributed to a corresponding period last year (from all sources) was £11,679 17s. 8d. During the past three weeks about £2,000 had been forwarded from the metropolitan workshops, making a total to date of nearly £13,500. The Council further reported that they had received information that a legacy of £300 had been left by the late Mrs. Evans to the London General Hospital Fund, and they had made a formal application for the same.

**Notices of Births, Deaths, and Marriages** are inserted in this column at a charge of 2s. 6d. for an announcement not exceeding four lines.

**Notice to Advertisers and Subscribers.**

All Advertisements, Orders for Copies of The Hospital and Business Communications should be addressed to **THE MANAGER, (Not to The Editor)**, The Hospital, 428, Strand, London, W.C.

The Cost of Subscription, post free, is as follows:—Per week, 2½d.; per quarter, 2s. 8d.; per half-year, 5s. 3d.; per annum, 10s. 6d. Foreign:—Per Annum, 15s. 2d.; payable, in all cases, in advance.

### NOTICE TO CORRESPONDENTS.

All MS., Letters, Books for Review, and other matters intended for the Editor should be addressed The Editor, The Lodge, Porchester Square, London, W.

The Editor cannot undertake to return rejected MS., even when accompanied by stamped directed envelope.

**"Burdett's Hospital Annual," 1895.**

Sixth year of publication. 950 pp. Scarlet cloth, gilt lettered. Price 5s. A most complete guide to British, Colonial, Indian, and American Hospitals, Dispensaries, Nursing and Convalescent Institutions, and Asylums. A few complete sets of the preceding five issues of the "Annual" may still be obtained on application to the Publishers, The Scientific Press (Limited), 428, Strand, London, W.C.

**NOTICE.—Vol. XVIII. of "The Hospital" (April to September, 1895), in handsome cloth binding, is now on sale at the Office of this Journal. Price 6s. Cases for binding the Numbers contained in this Volume, 1s. 6d. Index to Vol. XVIII., 2½d., post free.**

**The Monthly Part for October is now ready, price 6d.; by post, 8d.**

### SCRAPS AND GLEANINGS.

The Stockton Hospital Committee have a balance at the bank of £709 to the good.

A memorial tablet has been affixed to Professor Helmholz's house at Potsdam, where he was born in 1821.

The Goldsmiths' Company have sent a grant of £100 to Queen Charlotte's Lying-in Hospital, Marylebone Road.

Lord Rothschild has forwarded 20 pheasants to the Royal Hospital for Diseases of the Chest, City Road, for the patients.

Over £300 has been collected among workers at Dundee factories towards the income of the Royal Infirmary of that town.

A merchant of Havre, M. Kerdyeck, has just presented a large sum of money to the town for the endowment of a local convalescent home.

The executors of the late Mr. G. Lucas Callington have handed over a legacy of £90 from the deceased to the committee of the Plymouth Eye Infirmary.

The proceeds of a concert given by the Buxton Gardens Company's Orchestra have been handed over to the funds of the Devonshire Hospital and Buxton Bath Charity.

Lambeth workhouses are so overcrowded that more than 100 paupers have been transferred from the Lambeth Workhouse to that of the Risbridge Union in Suffolk.

The Salop Infirmary has received a sum of money from Mrs. Saxton, of Shrewsbury, for the purpose of endowing two beds in the children's ward in memory of her parents.

The sisters and nurses of the female side of the Greenwich Union Infirmary have presented Dr. W. J. C. Keate with a handsome dressing table set in tortoiseshell and silver.

It has lately been pointed out that the Hospital for Children, Great Ormond Street, which owes its existence to Charles Dickens, has nothing in the institution to commemorate the fact.

The Hackney Board of Guardians are maturing a plan to enter into negotiation with the Salvation Army authorities for the accommodation of their able-bodied paupers on the Hadley Farm Colony.

The trustees of the French Hospital, Shaftesbury Avenue, London, propose erecting a large convalescent hospital immediately east of Arundel Terrace, Brighton, on a vacant piece of land having an extensive sea front.

The Plague, during its recent outbreak in China, was the immediate cause of 2,550 deaths, and of the emigration of100,000 Chinese. The percentage of Europeans who recovered was 82, while that of Orientals was as low as 12.

An appeal is about to be made to the public by the Committee of the Morley House Convalescent Home, St. Margaret's Bay, Dover, for financial aid in carrying out the proposed extension work at that institution. About £3,000 is required for this purpose.

At the last meeting of the governors of the Middlesbrough Infirmary it was reported that the expenditure had increased, as compared with the corresponding period in 1894, but it was submitted that this was owing to the increased usefulness of the institution.

The friendly societies of Cirencester and Stratton made a special effort this month on behalf of the good work of the Cirencester Cottage Hospital. With this object a procession was formed for parading the town, and a service held in the parish church.

During the last six months legacies to the extent of £1,350 have been received at the convalescent house in connection with the Edinburgh Royal Infirmary. Among these an anonymous donation of £1,000, "In memory of Francis Brodie Imlach, F.R.C.S. Edinburgh," has been included.

A series of experiments, we learn, are being attempted in Utah, in the Territorial Reform School at Ogden, with the object of curing kleptomania and other such mental conditions in children, it being claimed that "Certain suggestions in the hypnotic state," will in all probability overcome criminal tendencies!

The Bishop of Coventry has instituted a charge for advertisement boards placed in St. Phillip's Churchyard. Hitherto placards relating to charitable collections have been allowed in the yard free of charge, but now the Hospital Sunday Collection Committee have had a fee of 4s. imposed upon them by the Bishop's orders.

Leprosy is, to all appearance, very much on the decrease in Norway. There were about 3,000 lepers in that country in 1856; in the latter end of 1892 there were only 900 cases. Dr. Kaurin's statistics recently published on this question show that a strict segration of lepers is important for the eradication of the disease.

The Medical Society of Berne has inaugurated a scheme, which it would be as well if other places would also adopt; this is the suppression of press notices of cases of suicide. It has been observed that suicides have so frequently been "suggested" by these notices that the measure seems a salutary one from this point of view.

The townspeople of Brighton are being attacked regarding the abuse of the medical charities there. Brighton is one of the richest communities in the kingdom, and yet we see by statistics that out of the 150,000 of its inhabitants 38,113 patients were last year admitted to the various hospitalsand dispensaries in the town.

In Berlin they are applying most strenuous measures, at a certain dairy, to preserve perfect purity in their milk. The managers have it so arranged that the milk is strained through a wire sieve with a cloth, on which there is a deep layer of finest sand. This sand is baked in a hot oven before it is again applied to the same method.

### The Book World of Medicine and Science.

### Books Received.

H. K. Lewis.

"Dental Materia Medica." By James Stocken.

"Elements of Medicine." By Dr. Carte.

**Periodicals and Pamphlets.**—Westminster Review, Humanitarian, Monthly Homœopathic Review, The Happy Home, Gleanings from Popular Authors, Part I., The Corpuscle, Cassell's Family Magazine, English Illustrated, Leisure Hour, Girl's Own Paper, Sunday at Home, Boy's Own Paper, Review of Reviews.

### The Student of Medicine.

### APPOINTMENTS.

L. Cane, M.D.Lond., B.S., appointed Physician to Peterborough Infirmary. J. MacNaughtan Christie, M.B., C.M.Glas., L.M. (Rot. Hosp. Dubl.), appointed an Honorary Surgeon to the New Plymouth Hospital, New Zealand. C. Cotton, F.R.C.P.Edin., appointed Physician to the Seaman's Infirmary and General Hospital, Ramsgate. J. G. Deacon, M.D., M.Ch., D.P.H., L.A.H., appointed Medical Officer for the No. 4 District of the Croydon Union. J. L. Fletcher, M.B., C.M.Edin., appointed Medical Officer of Health to the South Darley Urban District. T. A. Garrod, M.D.Edin., appointed Physician to the Newport and County Infirmary. J. Gilbert, L.R.C.S.I., L.M., L.A.H.Dubl., appointed Medical Officer for the Cruskeen Dispensary District. C. A. Hill, M.B., B.C., B.A.Cantab., M.R.C.S.Eng.L.R.C.P.Lond., appointed Obstetric Assistant to St. George's Hospital. J. H. Jones, appointed Medical Officer of Health to the Newport County Council. W. M. Parham, M.D.Edin., M.B., C.M., appointed Medical Officer for the Workhouse of the Brecknock Union. Dr. E. C. Pern, appointed Medical Officer of Health to the Droxford Rural District Council. J. N. Phillips, L.R.C.P.Lond., M.R.C.S.Eng., appointed Medical Officer of Health to the Cannock Urban District Council. W. Renton, L.R.C.P.Edin., L.R.C.S.Edin., L.M., L.S.A.Lond., appointed Clinical Assistant to the South London Royal Ophthalmic Hospital. S. E. Rigg, M.R.C.S., L.R.C.P., appointed House-Surgeon to the Royal United Hospital, Bath. W. A. Rutherford, M.B., C.M.Edin., late House-Surgeon to the Morpeth Dispensary, appointed House-Surgeon to the County Hospital, Durham. H. S. Taylor, M.R.C.S., L.R.C.P., appointed Senior House-Surgeon to the Clayton Hospital, Wakefield. St. C. Thomson, M.D., F.R.C.S., appointed Assistant Surgeon to the Royal Ear Hospital, Frith Street, Soho Square. S. Tippett, M.R.C.S., L.R.C.P., appointed Senior House-Surgeon to the Westminster Hospital. W. W. Walker, B.A., M.B., B.C.Cantab., appointed Assistant Physician to the Peterborough Dispensary and Infirmary. A. L. Webb, M.R.C.S., L.R.C.P., appointed Junior House-Surgeon to the Clayton Hospital, Wakefield. J. H. Wilson, M.D., B.S.Durh., appointed Medical Officer of Health for the Standish-with-Langtree Urban Sanitary District and Medical Officer for the Standish District of the Wigan Union.

### VACANCIES.

**The following vacancies are announced this week, the amount of salary in each case being given after the name of the institution:—**

### Resident Medical Officers.

Birmingham and Midland Free Hospital for Sick Children.—Resident Medical Officer and Resident Surgical Officer. Salaries £70 and £50 respectively, with board, washing, and attendance at the institution. Applications to the Secretary, Children's Hospital, Steelhouse Lane, Birmingham, by November 5th.

Carlisle Dispensary, Carlisle.—House Surgeon. Salary £130 per annum, with apartments, not board. Applications to Mr. G. A. Lightfoot, Honorary Secretary, 14, Bank Street, Carlisle, by October 29th.

Caterham Asylum.—Second Assistant Medical Officer under Metropolitan Asylums Board. Must not exceed 35 years of age, and duly registered and qualified to practise both medicine and surgery in England. Will be subject to annual election after the completion of his third year of office. Salary £120 per annum, rising £10 annually to £150. Forms of application may be obtained at the Offices of the Board, Norfolk House, Norfolk Street, Strand, W.C. Applications and testimonials to T. Duncombe Mann, Clerk to the Board, before October 30th.

General Hospital, Birmingham.—Assistant House Surgeon. Appointment for six months. No salary, but residence, board, and washing provided. Applications to Howard J. Collins, House Governor, by October 26th.

Hospital for Sick Children, Great Ormond Street, Bloomsbury. W.C.—House Physician and also House Surgeon; unmarried. Appointment for six months. Salaries £20 each, with board and residence in the hospital. Applications and testimonials to the Secretary before October 29th.

Hull Borough Asylum.—Assistant Medical Officer. Must be registered, unmarried, and not more than 30 years of age. Salary, £100 per annum, with board (no liquor), lodging, and washing. Applications and testimonials, endorsed "Asylum Medical Officer," to the Town Clerk, Town Hall, Hull, by October 30th.

North-West London Hospital, Kentish Town Road.—Resident Medical Officer and Assistant Resident Medical Officer. Appointments for six months. Salary at the rate of £50 per annum attached to the senior post. Applications to Alfred Craske, Secretary, by October 28th.

St. Marylebone General Dispensary, 77, Welbeck Street, Cavendish Square.—Assistant Resident Medical Officer; doubly qualified. Appointment for six months. Salary at the rate of £50 per annum, with furnished apartments, attendance, coal, and light. Applications to the Directors by October 31st.

### BOOKS FOR NURSES

Now ready, demy 8vo., blue buckram, gilt, 3s. 6d., with numerous illustrations.

**DIET IN SICKNESS AND IN HEALTH.** By MRS. ERNEST HART, Bachelier-ès-Sciences-ès-Lettres (restreint), formerly Student of the Faculty of Medicine of Paris, and of the London School of Medicine for Women. With an introduction by Sir Henry Thompson, F.R.C.S., M.B. Lond. A practical treatise on diet both in health and sickness, based upon the most recent scientific and medical knowledge; but written in popular and comprehensive language, and accompanied by a series of valuable recipes for the feeding of invalids and others.

Ready immediately, New and Enlarged Revised Edition. Profusely Illustrated with over 100 Cuts. Cloth, crown 8vo., price 3s. 6d., post free.

**THEORY AND PRACTICE OF NURSING:** A Text-Book for Nurses. By PERCY G. LEWIS, M.D., M.R.C.S., L.S.A. Always a standard work on Nursing, it now occupies the position of a classic, being used throughout the world in Training Schools and by Private Students. In order to bring this standard work thoroughly in line with the times, the author has most carefully revised it throughout, and to meet all demands has added chapters on Monthly Nursing and Confinements, consisting in all of some 50 additional pages. This Standard Manual may now therefore be taken as the most complete work of its kind in the market.

"We warmly recommend it as a nursing manual . . . full of useful hints and information."—*Birmingham Medical Review.*

NEW AND REVISED EDITION. Demy 8vo, 209 pp., profusely Illustrated with Copyright Portraits and Drawings. Price 2s. 6d., post free.

**HOW TO BECOME A NURSE: AND HOW TO SUCCEED.** A complete guide to the nursing profession for those who wish to become Nurses, and a useful book of Reference for Nurses who have completed their training and seek employment. Compiled by HONNOR MORTEN. Author of "Sketches of Hospital Life," "The Nurse's Dictionary," &c.

SECOND AND REVISED EDITION. Demy 16mo, suitable for the apron pocket, handsomely bound in cloth, 140 pp. Price 2s.; in handsome leather, gilt, price 2s. 6d. net.

**THE NURSES' DICTIONARY OF MEDICAL TERMS AND NURSING TREATMENT.** Compiled for the use of Nurses, by HONNOR MORTEN. Containing descriptions of the principal Medical and Nursing Terms and Abbreviations, Instruments, Drugs, Diseases, Accidents, Treatments, Physiological Names, Operations, Foods, Appliances, &c., &c., encountered in the Ward or Sick-room.

Demy 8vo, 200 pp., cloth extra. Price 3s. 6d.

**SURGICAL WARD-WORK AND NURSING.** A practical Manual of Clinical Instruction for Nurses and Students. This book will be found especially useful to beginners, to whom its simple description of elementary principles and treatment in surgery will render it invaluable. By ALEXANDER MILES, M.D. (Edin.), C.M., F.R.C.S.E. Over 100 Illustrations.

"Will furnish much-needed guidance to the student and the nurse in the early stages of their apprenticeship."—*Times.*

Third Edition. Enlarged and partly rewritten, cloth gilt, crown 8vo., about 200 pp., price 2s 6d, post free.

**HOSPITAL SISTERS AND THEIR DUTIES.** By EVA C. LUCKES, Matron to the London Hospital. The favourable reception accorded to the first two editions of this useful and interesting work, and the regular demand for copies of it, encourage the publishers to place this third edition, greatly improved and enlarged, before the Institutions. The experience and position of its author entitle it to authority, and every Matron, Sister, or Nurse who aspires to become a Sister should read the advice and information contained in its pages.

London: THE SCIENTIFIC PRESS (Limited), 428, STRAND, W.C.

### "THE HOSPITAL"

AN INDEPENDENT JOURNAL OF

**The Medical Sciences**

AND

**Hospital Administration,**

WITH WHICH IS ISSUED WEEKLY

A SPECIAL

### SUPPLEMENT FOR NURSES.

PUBLISHED EVERY SATURDAY.

PRICE TWOPENCE.

Matrons, Sisters, or Nurses will find in The Hospital a chronicle of the latest Nursing News, Practical Articles of the highest value to those who wish to keep themselves abreast with the times, and brightly written articles from Nurses all over the world.

SUBSCRIPTIONS CAN COMMENCE AT ANY TIME.

### TERMS OF SUBSCRIPTION.

### WEEKLY ISSUE.

| | Great Britain. | Foreign and Colonial. |
|---|---|---|
| For One Year ... | 10s. 6d. ... | 15s. 2d. |
| For Six Months | 5s. 3d. ... | 7s. 7d. |
| For Three Months... | 2s. 8d. ... | 3s. 10d. |

### MONTHLY PARTS.

| | Post Free to any Part of the World. |
|---|---|
| For One Year ... | ... 8s. 6d. |
| For Six Months ... | ... 4s. 3d. |
| For Three Months ... | ... 2s. 2d. |

Postal Orders and Cheques should be made payable to "The Scientific Press, Limited."

N.B.—In order to prevent delay, all business communications should be addressed to "The Manager" only, and not to any person individually.

### CHANGE OF ADDRESS.

In event of change of address, notice should be forwarded to the Publishers by the Saturday previous to date of publication, and the old address should also be given.

"The Hospital" can be obtained of all Booksellers and Newsagents in the United Kingdom, and at all the Railway Stalls, of Messrs. W. H. Smith and Son; and also from the following Agents in the Colonies and Abroad:—

Paris: Librairie Galignani, 224, Rue de Rivoli.

Berlin: Messrs. A. Asher and Co.

Leipsig: Mr. F. A. Brockhaus.

New York: The International News Co.

Montreal: The Montreal News Co.

Toronto: The Toronto News Co.

Melbourne, Sydney, Adelaide, and New Zealand: Messrs. Gordon and Gotch.

India: At all the Railway Bookstalls and Agencies of Messrs. A. H. Wheeler and Co.

South Africa: Messrs. Juta, Cape Town.

Publishing Offices: 428, Strand, London, W.C.

*In the Press. Price 5s.*

### BURDETT'S OFFICIAL NURSING DIRECTORY

A DIRECTORY, NOT A REGISTER.

The Hospital states: "It is well to bear in mind the difference and distinction which exists between a register and a directory. A register implies rights, and suggests that those who are not upon it are in some way or another inferior beings. But a directory is another matter. A directory gives no rights; insertion in it implies no adherence to any particular party or any particular opinion, and in no way interferes with entry on the register of any association, or the list of any institute, to which the individual may belong. All that a directory professes to do is to give information. The 'Medical Register' is not a mere list of qualified medical men; it is admission to the Register, which itself makes him qualified. For years, perhaps even now, 'Churchill's Medical Directory' contained the names of medical men who, so far as public appointments were concerned, were unqualified; it also contains the names of homœopathists, whom four out of every five medical men will not meet; and it certainly contains the names of many for whose moral character we should not like to vouch. But it gives full and accurate information about them all, and for the sake of that information it is constantly consulted both by the public and the doctors."

What Nurses Need.

"At present there is no Register for the nursing profession giving any legal status. Such a thing may come or it may not; but in either case there is no reason why there should not be a Directory, giving such information about the career of each nurse as both the public and the profession want; information, by the by, which no legal Register would be likely to insert. The 'Medical Register' is a bald and meagre thing compared with the 'Medical Directory.' What Burdett's 'Official Nursing Directory' proposes to do is to supply the want of a directory giving information. It will give no rights, it will imply no partisanship, it will give no guarantee except that what it prints is true, and is derived from official sources."

Acceptable to All Nurses.

"This ought to be enough to make it acceptable to the public, and every nurse ought to make sure that, whatever list or register she may be on, her name shall also appear on Burdett's 'Official Directory.'" Applications for forms should be made at once to the Editor, "Burdett's Official Nursing Directory," 428, Strand, W.C.

**The above work may be subscribed for at a reduced price by those whose names appear in the book, and who supply full particulars on a form which can be obtained upon application to the Publishers,**

THE SCIENTIFIC PRESS (Lim.), 428, Strand, London, W.C.

### NURSING BOOKS AT FULL DISCOUNT.

*LIST POST FREE.*

The Theory and Practice of Nursing (Percy Lewis, M.D.), 3/6 for **2/8.**
The Nurse's Dictionary (Honnor Morten), 2/- for **1/6.**
The Nurse's Case Book, 6d. for **3½d.**

### "THE HOSPITAL,"

AN INDEPENDENT JOURNAL OF
**THE MEDICAL SCIENCES AND HOSPITAL ADMINISTRATION.**

**Now ready, MONTHLY PART FOR OCTOBER, price 6d.,**

Containing interesting Articles on:—

The Respiration of Plants.

The Study of Man.

English Doctors Abroad.

On Sight-seeing.

On Modern Progress in Ophthalmic Medicine and Surgery. By R. B. Carter, F.R.C.S.

The Symptoms of Aneurisms. By A. Pearce Gould, F.R.C.S.

Drugs and Drink.

Medical Progress and Hospital Clinics.

Chloroform Fatalities.—III. By Sir B. W. Richardson, M.D., F.R.S.

Social Problems.

New Appliances & Things Medical.

Annotations on Various Subjects.

The Institutional Workshop.

The Book World of Medicine and Science.

**With Four Extra Nursing Supplements.**

Publishing Offices: 428, Strand, London, W.C.

